# The impact of midlife on migraine in women: summary of current views

**DOI:** 10.1186/s40695-020-00059-8

**Published:** 2020-10-06

**Authors:** Jelena M. Pavlović

**Affiliations:** 1 Albert Einstein College of Medicine, Department of Neurology, 1225 Morris Park Avenue, Van Etten 3C9B, Bronx, NY 10461 USA; 2 Montefiore Medical Center/Montefiore Headache Center, 1250 Waters place, 8th floor, Bronx, NY 10461 USA

## Abstract

Migraine is three times more common in women than in men and is the 4th leading cause of disability in women. Onset of migraine increases at menarche, with peaks in prevalence in the late 30s, and a rapid decline after menopause. While the prevalence is highest among women of childbearing age the frequency of headache and burden of migraine frequently worsens during midlife. Abundant population data suggest that hormonal factors may trigger headache attacks and influence onset and remission. The midlife worsening of migraine is attributed to hormonal fluctuations characteristic of the menopausal transition. Drops in estrogen presumably lead to increased migraine attacks at the time of menses as well as during the menopausal transition. During the menopausal transition, recommended approaches include both acute and preventive non-hormonal and hormonal options as well as behavioral approaches. Herein, is a brief review on the presentation of migraine in women across the lifespan, with special emphasis on midlife and the menopausal transition and implications for treatment.

Migraine is a complex central nervous system disorder which is primarily characterized by headaches of long duration, with moderate to severe pain and associated symptoms such as light and sound sensitivity (photo- and phonophobia), nausea and vomiting [1]. More than “just a headache” it is a disorder of central nervous system dysregulation [2], that is three times more prevalent among women than men, with a cumulative lifetime prevalence among women of 43% [3]. It has long been recognized that migraine in women fluctuates with both monthly and life-stage dependent natural hormonal changes [4–6]. Though the peak prevalence of migraine occurs during the childbearing years, migraine symptomatology often worsens in midlife due to the hormonal fluctuations of the menopause transition, often with an increase in the number of headache days per month [7, 8]. This increase in migraine burden combined with the peak prevalence of migraine in the 4th and 5th decades of life, make migraine disease a major source of pain in midlife women. Although the increased burden of migraine among middle aged women has long been recognized in clinical practice, the epidemiology of migraine as women traverse midlife has been largely unexplored [9–11].

While there are abundant data on migraine during women’s reproductive years, there has been a paucity of studies focusing on migraine across the menopausal transition. Clinical practice has long held the notion that migraine improves in menopause, and this has been supported by the epidemiological studies which show migraine prevalence peaking in late 30s and declining thereafter [3, 12–14]. However, these data are based primarily on cross-sectional comparisons of migraine prevalence by age. Furthermore, given that migraine is a chronic disorder with episodic manifestations [15], it’s burden (impact on health, social, familial, occupational and academic life of an individual) varies across life span as the frequency and severity of attacks as well as the responsiveness to treatment and recovery between attacks fluctuate [16]. According to the Global Burden of Disease study, migraine is the 2nd most globally disabling condition after low back pain [17]. Though migraine burden tends to be the highest among people from economically marginalized communities, migraine remains underdiagnosed and undertreated in general and in women from marginalized communities in particular [18, 19]. To date, the prevalence of migraine within particular stages of the menopausal transition has not been well characterized, as large migraine epidemiological studies generally do not include detailed characterization of menopausal stage [8]. Furthermore, little is known about potential mechanisms by which changes in sex hormones impact patterns of migraine occurrence during the menopause transition and the eventual remission of migraine post-menopause. This review will examine migraine in women with a focus on hormonal influences that affect migraine, current understanding of how migraine occurrence changes in midlife, the implications for treatment and directions for future research.

## Migraine subtypes

The two primary types of migraine are migraine without aura which accounts for the majority of cases (approximately 80%) and migraine with aura which accounts for about 20% [1, 20]. Of the two, migraine without aura is the type primarily associated with hormonal fluctuations, and thus is the primary focus of this review (Table 1) [21, 22].

**Table 1:**
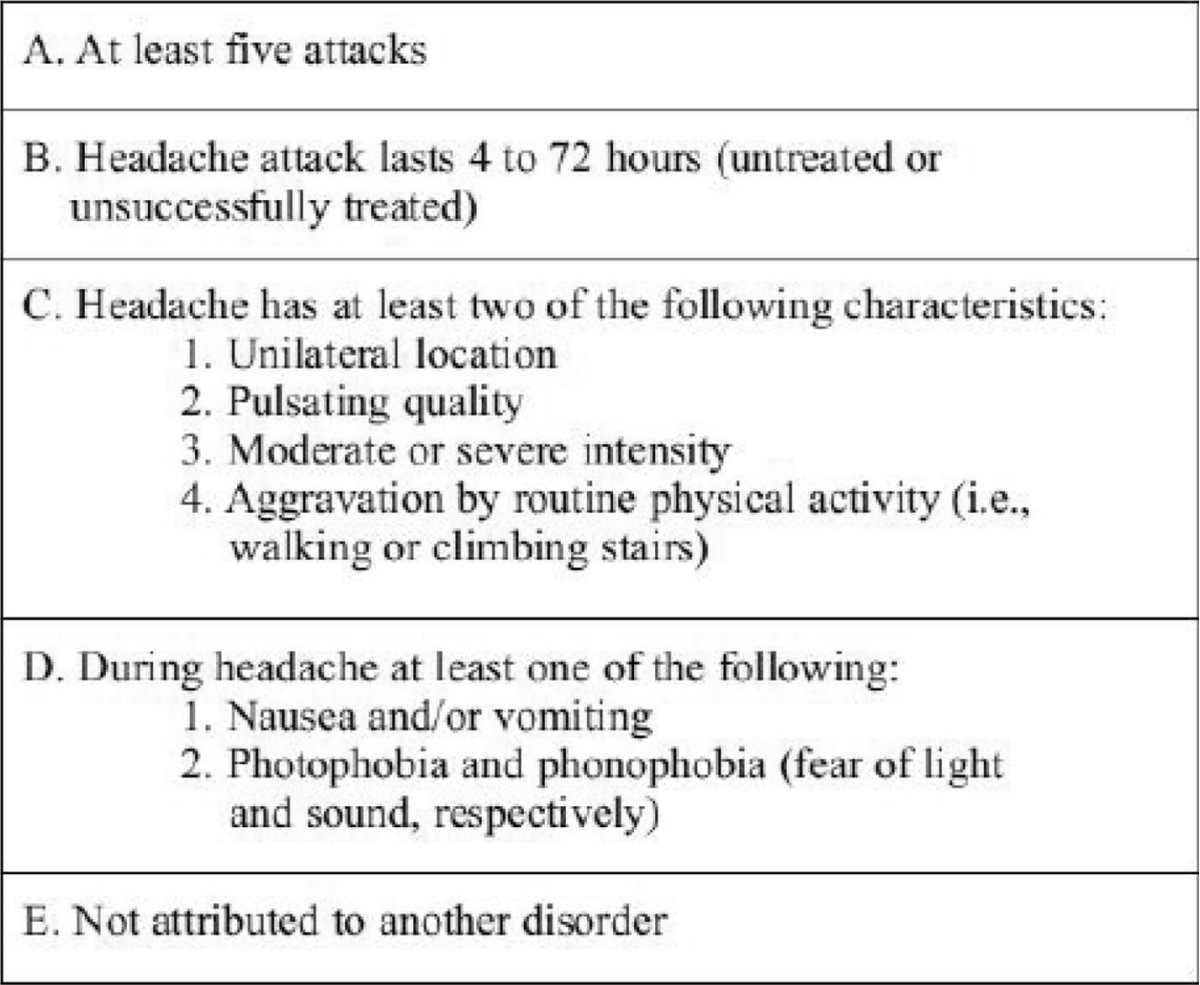
Diagnostic Criteria of Migraine Without Aura

Among women, migraine can be further subdivided with respect to its relationship to menstruation (Table 2) [1, 21]. Peri-menstrual migraine attacks are headache attacks that occur within the 5 day window spanning the 2 days prior to the onset of menstruation to the third day of menstruation (referred to as − 2 to + 3 in the migraine literature). Women who experience only peri-menstrual migraine attacks are categorized as having Pure Menstrual Migraine (PMM). This is a relatively rare condition which affects 7–12% of women with migraine who are of reproductive age [23]. In contrast to PMM, the majority of women with migraine experience migraine attacks both peri-menstrually and at other times of the month. This condition is defined as Menstrually Related Migraine (MRM), and is estimated to affect 50–70% of reproductive age women [23, 24]. Clinically, distinguishing PMM from MRM requires that the peri-menstrual headache attacks occurred during at least 2 out of the last 3 periods [1]. Finally, women who do not experience attacks in relation to menstruation are classified as Non-Menstrual Migraine.

**Table 2:** Diagnostic criteria for Menstrual Migraine

International Headache Society (ICHD-3beta) (Headache Classification, 2013) Diagnostic Criteria	
Pure menstrual migraine	Documented and prospectively collected evidence that attacks occur **exclusively** on day 1 ± 2 (i.e., days −2 to + 3)^a^ of menstruation in at least two out of three menstrual cycles and at no other times of the cycle
Menstrually-related migraine	Documented and prospectively collected evidence that attacks occur on day 1 ± 2 (i.e., days −2 to + 3)^a^ of menstruation in at least two out of three menstrual cycles and **additionally** at other times of the cycle
Non-menstrual migraine	Attacks have no menstrual relationship

^a^The first day of menstruation is day 1 and the preceding day is day − 1; there is no day 0

These subclasses of migraine related to menstruation are of significant interest as they can inform both pathophysiology and treatment. However, they have been insufficiently explored in population-based studies [8]. Much work is needed to improve understanding of the hormonal influence on migraine presentation and these subtypes. The need for additional information is highlighted by the fact that these subclassifications have not been fully incorporated into the International Classification of Headache Disorders (ICHD) clinical criteria, with menstrually related subtypes included only with the Appendix of the published diagnostic criteria [1].

## Migraine and hormones

After stress, menses is the most commonly reported migraine trigger [25–27]. The recognition of a 5-day window of migraine occurrence around the start of menses is predominantly based on women’s frequent reports that menstruation triggers their migraine attacks. This is further supported by clinical data [6, 28] and by experimental research from the 1970s that implicates estrogen “withdrawal” in the late luteal phase as the precipitant of menstrual migraine attacks [29, 30]. Although this link between falling estrogen levels and migraine attack occurrence has been recognized for over 40 years, relatively few studies have directly examined the role of specific sex hormone changes over the menstrual cycle in relation to migraine [6, 31–34]. The few available studies have been limited by small sample size (including less than twenty subjects) and have focused on comparisons of mean hormone levels for women with different subtypes of migraine [31, 32, 34]. In general, studies have lacked well characterized daily hormone cycles. None of the studies to date have explored longitudinal associations between patterns of hormone changes over the menstrual cycle and headache occurrence.

To compare patterns of ovarian hormone changes over the menstrual cycle for women with migraine and controls, we recently examined daily urinary concentrations of estrogen, progesterone, luteinizing hormone, and follicle stimulating hormone over the menstrual cycle for women enrolled in the Study of Women’s Health Across the Nation (SWAN) Daily Hormone Study [35]. These women were entering midlife with an average age of 47 years. Women with migraine history were observed to have a 30% more rapid rate of estrogen decline than controls, specifically within the late luteal phase of the menstrual cycle. This difference was independent of whether women in the migraine group experienced a headache in the cycle studied [35]. This suggested a “two-hit” hypothesis of peri-menstrual migraine attack triggering such that among women with history of migraine, more rapid estrogen decline following the late luteal peak may confer neuroendocrine vulnerability that facilitates initiation of migraine attack(s) by common triggers such as stress, disrupted sleep, foods, wine, etc. [25, 26].

The hypothesis that there is an endogenous difference in patterns of estrogen change in women with migraine is further supported by the results of a small study of postmenopausal women where a single injection of estradiol was administered. Following post injection estrogen withdrawal, migraine occurred only in women with a premenopausal history of peri-menstrual migraine attacks [36]. Combined, these studies suggest that neuroendocrine vulnerability in women with migraine history may play a role in the well-recognized but little understood hormonal regulation of the disorder. The relation of migraine to hormone fluctuations is likely complex and further studies are needed. For example, migraine attacks are generally stabilized during states of stable and high estrogen (as in the 2nd and 3rd trimester of pregnancy) [37] and exogenous estrogens have been effectively used to stabilize migraine in women with menstrually related attacks [24]. However, in contrast, treatment with exogenous estrogens has been associated with more frequent migraine attacks or even a precipitation of new onset migraine in some women [38].

It is likely that there may be different pathophysiologic mechanisms playing a role in menstrual vs non-menstrual attacks and that these may vary within women who are sensitive to hormonal fluctuations. Though the pathophysiology of these attacks is currently unknown, population-based studies have shown that women who experience peri-menstrual migraine attacks tend to have a higher burden of migraine than those with non-menstrual migraine [39]. Clinic-based studies support the commonly held notion that peri-menstrual migraine attacks are more severe, longer-lasting and refractory to both acute and prophylactic treatment and therefore substantially more burdensome than non-menstrual attacks [40–43]. .Peri-menstrual migraine attacks may have a typical duration of several hours, but in some cases head pain can last several days and may be extremely severe and poorly responsive to analgesics [40, 41, 43]. Though more burdensome, the predictability of peri-menstrual attacks in many women of reproductive age allows for effective short-term preemptive treatments that minimize duration of exposure to medication [44].

## Migraine during the menopausal transition

Although most women with migraine develop the disorder in their teens or twenties, 8–13% of women with migraine report the new onset of migraine during perimenopause [8]. In general, migraine is commonly under diagnosed in the population [45]. Thus, although some cross-sectional studies suggest that prevalence of migraine increases as women progress through the MT [8, 46], this may be due to increased ascertainment among women who present to the health care system with migraine when the frequency/burden reaches a critical threshold in perimenopause [7]. In a cross-sectional community based study of 1436 Taiwanese women aged 40–54, prevalence of migraine was similar in the premenopausal and early perimenopausal groups (16.7%) and significantly higher in the late perimenopausal group (31%) [46]. Data from the cross-sectional American Migraine Prevalence and Prevention study showed that among 3664 women with migraine (mean age 46), the risk of high frequency headache (defined as > 10 headache days per month) was significantly greater in the peri-menopause and early post-menopause stages compared to pre-menopause [7]. Though the factors affecting these differences across menopausal stages are not entirely understood, it has been shown that women in whom migraine frequency is exacerbated by hormones and associated with pre-menstrual syndrome, appear to have the best prognosis post-menopause, often experiencing complete resolution.

The few available epidemiological studies suggest that the increase in frequency and severity of migraine attacks in peri-menopause are due to peri-menopausal hormonal fluctuations [7, 8]. The limited data available suggest that more frequent estrogen withdrawal in peri-menopause is the primary driver of increased headache frequency during the menopausal transition. However, understanding of migraine pathophysiology in the menopausal transition has been limited as no longitudinal study to date has systematically examined hormonal changes across menopausal stages in relation to frequency of migraine/headache days. Prior studies have lacked longitudinal endogenous hormone data, detailed characterization of menopausal stage, and reliable migraine data. In particular, the largest prior studies are based in menopause clinics which have high quality hormonal data but which lack the criteria necessary for specific diagnosis of migraine. Due to these limitations, most of the current literature is based on comparisons of pre- and post-menopausal women with no longitudinal data on the trajectories of migraine across the menopausal transition. Furthermore, existing cross-sectional studies have been primarily based on retrospective headache questionnaires that do not accurately capture the fluctuations in headache over time.

In addition, the worsening of migraine in perimenopause may be due to increases in other conditions that are co-morbid with migraine. Midlife women are at increased risk of anxiety, depression and sleep disturbances [47–51], which are known to interact with migraine in synergistic ways, adding to the overall burden of the disorder. Stress, in particular, has a well-recognized and complex association with migraine. It is the most commonly recognized migraine trigger often precipitating attacks which in turn can lead to more stress resulting in even more attacks and possible transformation from episodic to chronic migraine [25, 26, 52].

Further, in cross-sectional studies, vasomotor symptoms have been reported to be more common among women with migraine [46]. A recent study using the SWAN cohort evaluated the presence of vasomotor symptoms in women with migraine over up to 10 years of follow-up [53]. This study found that a history of migraine predicted higher frequency of hot flashes and night sweats [53]. Overall, these studies support the notion that the prevalence and burden of migraine are most pronounced in perimenopause when hormonal imbalance is present, particularly in women with history of menstrual migraine.

The prevalence of migraine apparently declines after menopause with stabilization and lowering of endogenous estrogen levels. However, the post-menopausal course of migraine depends on whether menopause is natural or surgically induced [8]. While migraine improves in the majority of women following natural menopause, it worsens in 67% of women with surgical menopause [54]. Furthermore, in cases of menopause induced pharmacologically by a gonadotropin-releasing hormone agonist, an overall worsening of migraine has been observed [55]. In these women, treatment with estrogen improved headache pain severity but not the headache frequency. This may be in part due to increased stress in the setting of the procedure, leading to activation of migraine attacks. While medical oophorectomy has been suggested as treatment for women with severe chronic migraine, reports of the benefits of this approach are anecdotal and there is no systematic evidence to support this drastic approach.

Of note is that migraine prevalence also increases in transgender women using exogenous estrogen treatment and reduces in transgender men using testosterone. Importantly, migraine with aura can arise de novo in transwomen, presumably due to high doses of estrogens [56, 57].

## Treatment of Migraine during menopausal transition

Treatment of migraine in any phase of life may be acute or preventive. Though the majority of standard migraine treatments are indicated in women regardless of the menopause transition stage, the MT stage and co-morbid disorders and symptoms may impact the choice of treatment. In addition, clinicians should consider the characteristics and frequency of headaches, presence of other co-morbidities and medications and patient preferences. An integrated approach that includes non-pharmacologic strategies should be advocated with women encouraged to get regular adequate sleep, avoid skipping meals, engage in regular exercise, drink plenty of fluids, and avoid caffeine, tobacco and alcohol. Symptomatic nonpharmacologic treatments, such as breathing exercises, relaxation, and massage can also be recommended. Though time and resource consuming, formal relaxation training and thermal or EMG biofeedback training, is often appropriate and needed in conjunction with medication therapy [58].

Nonsteroidal anti-inflammatory drugs (NSAIDs) are the mainstay of acute treatment and can be combined with anti-emetics if necessary. Triptans are also used as the first line of acute treatment provided the patient does not have significant cardiovascular risk factors.

Mini-prevention techniques recommended for menstrual migraine (treating peri-menstrual migraines with NSAIDS and/or triptans) [59] in those with regular cycles often become unavailable to women with irregular cycles. This necessitates prevention with daily migraine medication, rather than more focused, intermittent prevention timed to the menstrual cycle. Preventive options for peri-menopausal women do not require special consideration, as most migraine preventive agents can be used, including anti-epileptics (topiramate and valproate), beta-blockers (propranolol), tricyclic anti-depressants (amitriptyline and nortriptyline). The new drug class of monoclonal CGRP antibodies developed specifically for migraine prevention provides a promising option for midlife women as it is well tolerated, easy to adhere to and not known to have major adverse or side effects [60].

An important consideration for migraine treatment in midlife women is that women traversing the menopausal transition may experience additional forms of pain and symptoms for which they may take daily analgesic medications [50, 61, 62]. It is important to assess these symptoms and their treatment, as the consumption of acute symptomatic drugs (such as NSAIDs, opioids, etc..) can further complicate migraine presentation by inducing a medication overuse headache (MOH) which can contribute to the chronification process and development of chronic migraine [63]. Treatment of transwomen is same as those of ciswomen.

In addition to commonly used acute and preventive medications that are used in both pre- and post- menopausal women, hormone therapy (HT) has been used in treatment of migraine during perimenopause in order to mitigate fluctuating estrogen levels [8, 64, 65]. This is generally limited to women with migraine without aura as exogenous estrogen use is generally not advised in women who have migraine with aura due to increased stroke risk [66, 67]. Furthermore, exogenous estrogen has been observed to trigger aura “de novo” or worsen the severity and frequency of preexisting attacks in women who have migraine with aura. Interestingly, a large cross-sectional population-based study in Norway examined the use of hormone therapy (HT) and headache in postmenopausal women and found that migraines and other headaches were more likely to occur among the post-menopausal women using HT than in those not using HT [65]. Due to the cross-sectional nature of this study, it cannot be determined if HT caused migraines or was used because of them. Overall, if estrogen stabilization is needed, it is recommended that peri-menopausal women with migraine be treated via the transdermal route of E2 administration at the lowest effective dose.

## Conclusion and future directions

Migraine is significantly affected by fluctuating sex hormone levels in women during menses and across the menopause transitions. While migraine generally tends to improve post-menopause, perimenopause can be associated with significant worsening in frequency and symptoms presumably due to fluctuating estrogen levels. The exact mechanism by which migraine worsens in midlife remains unknown as no study to date has focused on specific sex hormone changes over the menopausal transition in relation to migraine. Also unclear is the extent to which a history of menstrual migraine may impact the experience of migraine over the menopausal transition. Further, the complex way in which exogenous hormones and withdrawal from their use may impact migraine in women with various subtypes of migraine remains unclear, though there are likely to be substantial differences based on migraine subtype. The burden of migraine in midlife is further impacted by the fact that perimenopause provides treatment challenges due to the loss of predictability of hormonally related attacks and the worsening of symptoms that frequently occurs. Better understanding the relationship between both endogenous and exogenous hormones and migraine throughout midlife is of great importance given the disability and morbidity associated with migraine among midlife women. This increased understanding has potential to improve treatment and therefore overall burden of migraine.

## Data Availability

Not applicable.

## Abbreviations

ICHD: International Classification of Headache Disorders
HT: Hormone therapy
MRM: Menstrually Related Migraine
PMM: Pure Menstrual Migraine
